# Aptamer-based analyses of plasma proteome in individuals with post-COVID condition who underwent tailored physical activity

**DOI:** 10.3389/fspor.2026.1797346

**Published:** 2026-05-29

**Authors:** Mohammad Mobarak H. Chowdhury, Marie-Noelle Fontaine, Sarah-Eve Lord, Jean-François Lucier, Hugues Allard-Chamard, Subburaj Ilangumaran, Alain Piché, Isabelle J. Dionne, Sheela Ramanathan

**Affiliations:** 1 Department of Microbiology and Infectious Diseases, Faculty of Medicine and Health Sciences, Université de Sherbrooke, Sherbrooke, QC, Canada; 2 Faculty of Physical Activity Sciences, Université de Sherbrooke, Sherbrooke, QC, Canada; 3 Department of Biology, Faculty of Science, Université de Sherbrooke, Sherbrooke, QC, Canada; 4 Department of Medicine, Faculty of Medicine and Health Sciences, Université de Sherbrooke, Sherbrooke, QC, Canada; 5 Department of Immunology and Cell Biology, Faculty of Medicine and Health Sciences, Université de Sherbrooke, Sherbrooke, QC, Canada; 6 Research Centre on Aging, Affiliated with CIUSSS de l'Estrie-CHUS, Sherbrooke, QC, Canada

**Keywords:** aptamer, long COVID, plasma proteomics, post-COVID condition, SomaScan, tailored physical activity

## Abstract

**Introduction:**

Post-COVID condition (PCC) encompasses a spectrum of clinical symptoms affecting multiple organs that persist for >3 months after resolution of the initial SARS-CoV-2 infection. The complex nature of PCC symptoms makes it difficult to decipher the mechanistic basis of disease and establish efficient pharmacological interventions. However, non-pharmacological approaches such as personalized physical exercise regimen has been shown to improve the quality of life in PCC patients. Among the non-pharmacological interventions for PCC, physical rehabilitation, especially symptom-titrated physical exercise, has shown promise in restoring the quality of life but the underlying mechanisms are not yet elucidated. This personalized approach adjusts the physical training intensity according to patient's ability and tolerance levels.

**Methods:**

We had observed that individuals with PCC showed significant improvement following tailored physical exercise. Here the plasma samples obtained before and after the intervention was analyzed for a preselected panel of 1500 markers by SomaScan assay.

**Results:**

Proteins differentially expressed between the exercise and no-exercise groups suggest that the tailored exercise training drives distinct proteomic signatures involved in immune, vascular and oxidative stress pathways.

**Discussion:**

Despite the low sample numbers, our results indicate that the tailored exercise regimen resulted in significant alterations in biological pathways associated with PCC.

## 1 Introduction

Post-COVID Condition (PCC), also referred to as Long COVID, has emerged as one of the most significant long-term public health challenges following the global SARS-CoV-2 pandemic. PCC is characterized by a broad spectrum of persistent symptoms, including fatigue, exercise intolerance, dyspnea, cognitive dysfunction, sleep disturbances, and autonomic irregularities that continue for weeks to months after resolution of the acute infection. Evidence increasingly suggests that PCC involves a complex interplay of ongoing immune activation, mitochondrial dysfunction, redox imbalance, endothelial injury, and metabolic dysregulation (1, 2). Despite the growing body of literature describing PCC mechanisms, sensitive molecular biomarkers capable of characterizing disease state and monitoring therapeutic response remain limited.

Physical activity has been widely recognized as a powerful physiological modulator with systemic effects on immunity, metabolism, mitochondrial biology, and antioxidant defense (3–8). Advances in high-throughput proteomics have shown that exercise induces dynamic remodeling of intracellular and extracellular protein networks, including modifications to proteins involved in oxygen transport, mitochondrial oxidative phosphorylation, redox balance, immune regulation, and ATP production (9–12).

Several lines of evidence also suggest that tailored physical activity can improve the quality of life in selected individuals with long COVID (13–15). Epidemiological and clinical studies indicate that regular physical activity is associated with improved symptom recovery, reduced hospitalization duration, and enhanced immune resilience in patients recovering from COVID-19. We were the first group to show that certain proteins involved in oxidative stress were differentially regulated in the plasma proteome of individuals with PCC who underwent a tailored physical activity regimen (13). Nonetheless, the molecular impact of structured exercise interventions on PCC remains insufficiently characterized.

High-throughput proteomic technologies provide an essential framework for uncovering molecular fingerprints associated with exercise responsiveness in plasma samples. Mass spectrometry-based data-independent acquisition (DIA-MS) offers deep proteome coverage, unbiased peptide selection, and robust quantification suitable for detecting subtle physiological changes. In parallel, aptamer-based platforms such as SomaScan® utilize Slow Off-rate Modified Aptamer (SOMAmer®) reagents to measure thousands of proteins simultaneously. SOMAmer reagents recognize conformational epitopes with high affinity and specificity, enabling large-scale proteomic screening with excellent reproducibility (16). However, because DIA-MS detects proteins based on peptide ionization and fragmentation, whereas SomaScan relies on structural epitope recognition, these technologies interrogate different regions of the plasma proteome. Consequently, cross-platform differences are common, and proteins detected by one method may not be detected by the other due to epitope accessibility, post-translational modifications, or abundance thresholds.

In our previous study, we employed DIA-MS–based plasma proteomics to investigate the molecular effects of an 8-week tailored exercise regimen in PCC patients (13). In this study, we analyzed the same plasma samples by SomaScan's high-throughput aptamer-based proteomics to identify/validate key biomarkers discovered in the DIA-MS study but also uncover additional PCC-specific proteins and pathways.

## 2 Methodology

The study population and intervention framework have been previously described in detail in Chowdhury et al. (13). In brief study participants with clinically diagnosed PCC were randomly assigned to exercise (*n* = 15, 7 men and 8 women) or no-exercise (*n* = 10, 2 men and 8 women) groups. No significant differences were observed in the baseline physical, anthropometric and clinical parameters between the two groups (13). The most frequent symptoms reported were fatigue, sleep disturbance, dizziness, chest pain, arrhythmia, dyspnea, cough, abdominal pain, diarrhea, joint pain, myalgia, skin rash, anxiety and depression, headache, loss of taste or smell and brain fog. All the participants were able to complete the training protocol and none of them reported post-exertional malaise in this study (13). The exercise group underwent supervised training in the laboratory setting for 12 weeks in addition to standard care. The no-exercise group received standard care only. The exercise group engaged in three non-consecutive mixed exercise sessions per week lasting less than one hour, that were conducted at the same hour of the day (13). The training sessions were tailored to the capacity of each participant and was graded on the Borg scale (13). Resistance training was alternated with endurance and were carried out in the presence of trained kinesiologists in a research gym setting (13). Non-fasting plasma samples were collected at the beginning and end of the intervention in the morning and were immediately aliquoted and stored at −80 °C as described (13). For the present analysis, pre- and post-intervention plasma samples from the exercise and no-exercise groups were analyzed using the SomaScan® aptamer-based platform to validate DIA-MS–identified biomarkers and explore additional PCC-associated protein signatures.

### 2.1 SomaScan® assay

Profiling of plasma proteome was carried out using the SomaScan® Assay, which is a highly multiplexed aptamer-based proteomic technology. In short, the technology is based on proprietary Slow Off rate Modified Aptamers (SOMAmer reagents) selected to bind to structural epitopes on proteins. During synthesis, a Cyanine-3 fluorophore, spacer, and photocleavable biotin are incorporated at the 5' end of each SOMAmer reagent. These SOMAmer reagents are pre-immobilized onto streptavidin beads and used to capture target proteins from biological samples during an incubation step. Unbound proteins are washed away, and captured proteins are biotinylated using NHS-biotin. UV light is used to cleave the photocleavable linker, releasing complexes back into solution in the presence of a high concentration of universal polyanionic competitor. This step takes advantage of solution kinetics to selectively enrich for specificity. Fast off-rate, non-specific complexes dissociate, and rebinding is blocked by the competitor. Slow off-rate, specific complexes do not dissociate on this timescale. Complexes and some free proteins that dissociated are captured onto new streptavidin beads. These dissociated proteins do not contribute to signal downstream since they are no longer bound to a SOMAmer reagent. After washing, SOMAmer reagents are eluted from the beads by denaturing the proteins with a chaotropic salt, sodium perchlorate. The eluate is placed onto a custom Agilent microarray with probes complimentary to each SOMAmer reagent for overnight hybridization. Slides are washed and read in an Agilent microarray scanner. Supplementary Document S1 provides the quality control metrics reported by the SomaScan platform. The resulting RFU values correlate to the amount of target epitope in the initial samples. Data was obtained for 1,500 proteins, which were selected based on the following panels—Cardiovascular Disease Panel, Inflammation and Immune Response Panel, Metabolic Diseases Panel, Respiratory Panel, Cytokines Panel, Neuroscience Panel and Oncology Panel, and proteins identified to be differentially expressed in PCC [https://covimapp.serve.scilifelab.se/; (Chowdhury, 2026 #165)]. Custom R scripts were generated by Mr. JF Lucier (Bioinformatics platform) to analyze the Somascan data. A custom python script was developed to preprocess SomaScan proteomics data adat file. The SomaDataIO R package reads the adat file, merge with clinical/sample metadata, filters to remove calibration or buffer controls and exports clean tables for downstream statistical analysis. Custom python script was used to generate proteins stats like group average, group STDEV, log2fold change, t-test *p* values and adjusted *p*-value.

### 2.2 Statistical analyses

Statistical analyses conducted using GraphPad Prism version 10.0.3 (San Diego, CA, USA). GraphPad Prism was employed to create volcano plots and quantify protein abundance between the study groups. Venn diagrams were generated using the jvenn online tool (https://jvenn.toulouse.inrae.fr/app/index.html). GO pathway analysis using R library pathfinder (https://github.com/egeulgen/pathfindR). Default parameter values were used for all analyses. For pval thresholds and enrichment thresholds, default values are: *p*_val_threshold the *p* value threshold to use when filtering the input data frame. Must be a numeric value between 0 and 1. (default = 0.05) enrichment_threshold: adjusted-*p* value threshold used when filtering enrichment results (default = 0.05). Gene ontology analysis and Heatmaps were generated with the SRplot web server (https://www.bioinformatics.com.cn/en).

## 3 Results

In our study (13) we addressed the changes in physiological parameters following symptom-titrated physical exercise in conjunction with unbiased global analyses of plasma proteome by proteomics. In individuals living with PCC for more than 6–12 months certain biomarkers, such as CST3 were downregulated after the exercise regimen (13). Additional markers of inflammation such as S100A8/9 and LYZ, that were shown to be associated with COVID-19 in our study (17) and from other studies (18, 19) were also downregulated following the training program (13).

**Figure 1:**
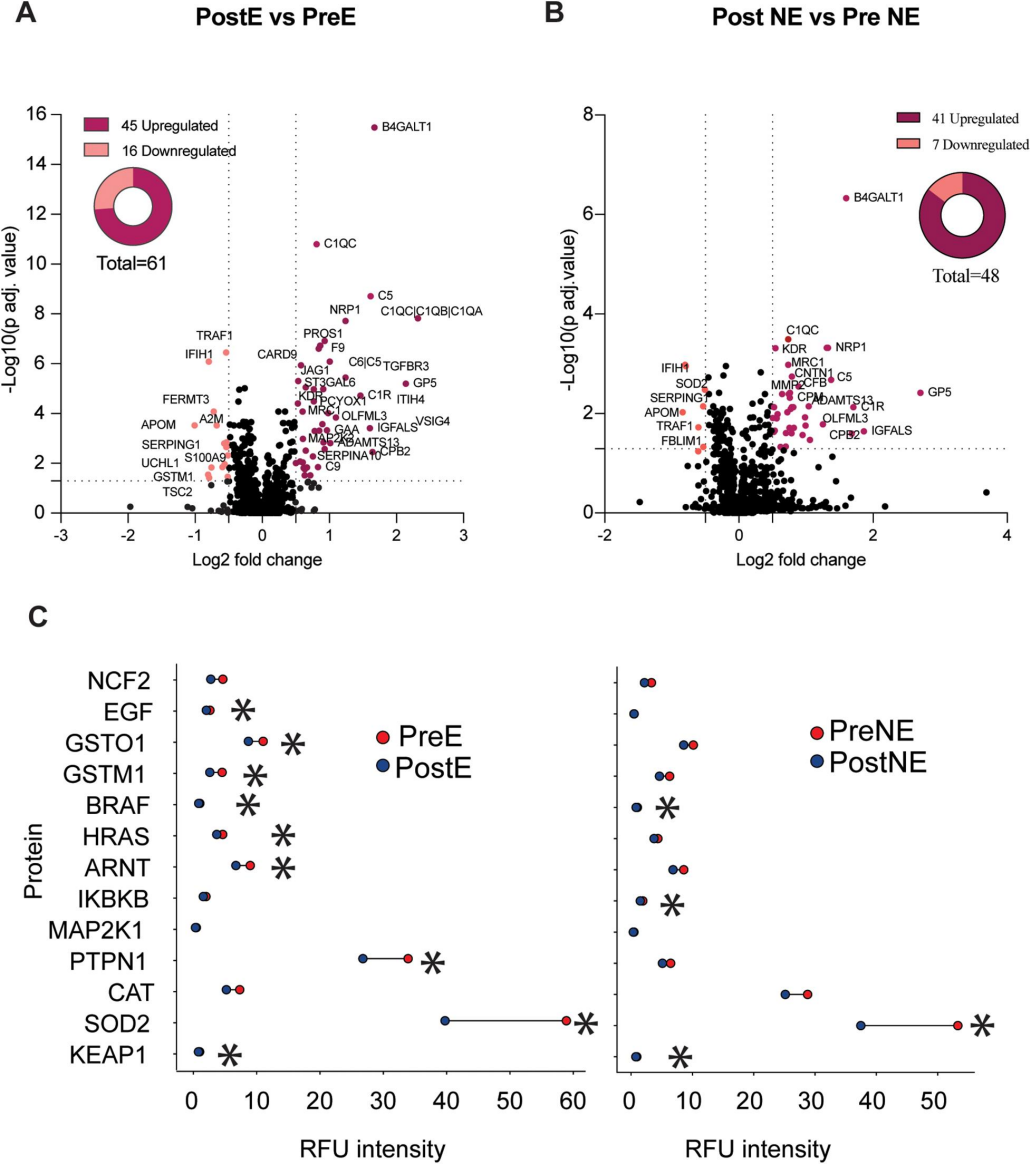
Differentially expressed proteins and functional enrichment following exercise regimen in PCC participants. Volcano plot comparing protein expression between **(A)** post-exercise (PostE) and pre-exercise (PreE) (*n* = 15) and **(B)** post no-exercise (PostNE) and pre no-exercise (PreNE) (*n* = 10) groups. **(C)** Plasma protein levels were quantified using SomaScan proteomics). * Adjusted *p* value <0.05. were plotted. RFU, relative fluorescence units.

In the current study, we analyzed the plasma samples from the above study for 1,500 proteins using Somascan technology. The analytes were defined by the SomaScan assay panel used (SomaLogic Inc.). The panel was designed by SomaLogic Inc. to cover diverse biological processes, including immune and inflammatory pathways, cardiovascular function, and metabolism, with many already reported in COVID-19 and long COVID studies (Supplementary Table S1). Among the 1,500 proteins analyzed, proteins which showed an adjusted *p* value > 0.05 (Log2 fold cut range 0.5 to −0.5) were considered as differentially expressed and were included in the analyses. Comparisons were made between post-exercise (PostE) and pre-exercise (PreE) samples and the no-intervention (no-exercise NE) groups. As the samples were obtained from the same individuals over at an interval of 12 weeks, the differentially expressed proteins were lower in numbers (Figures 1A,B). The volcano plot demonstrates robust changes, with several proteins reaching high statistical significance (adjusted *p* < 0.05). Some proteins were significantly modulated independent of the exercise regimen, while some were specific to the exercise intervention group (Figure 1C).

**Figure 2:**
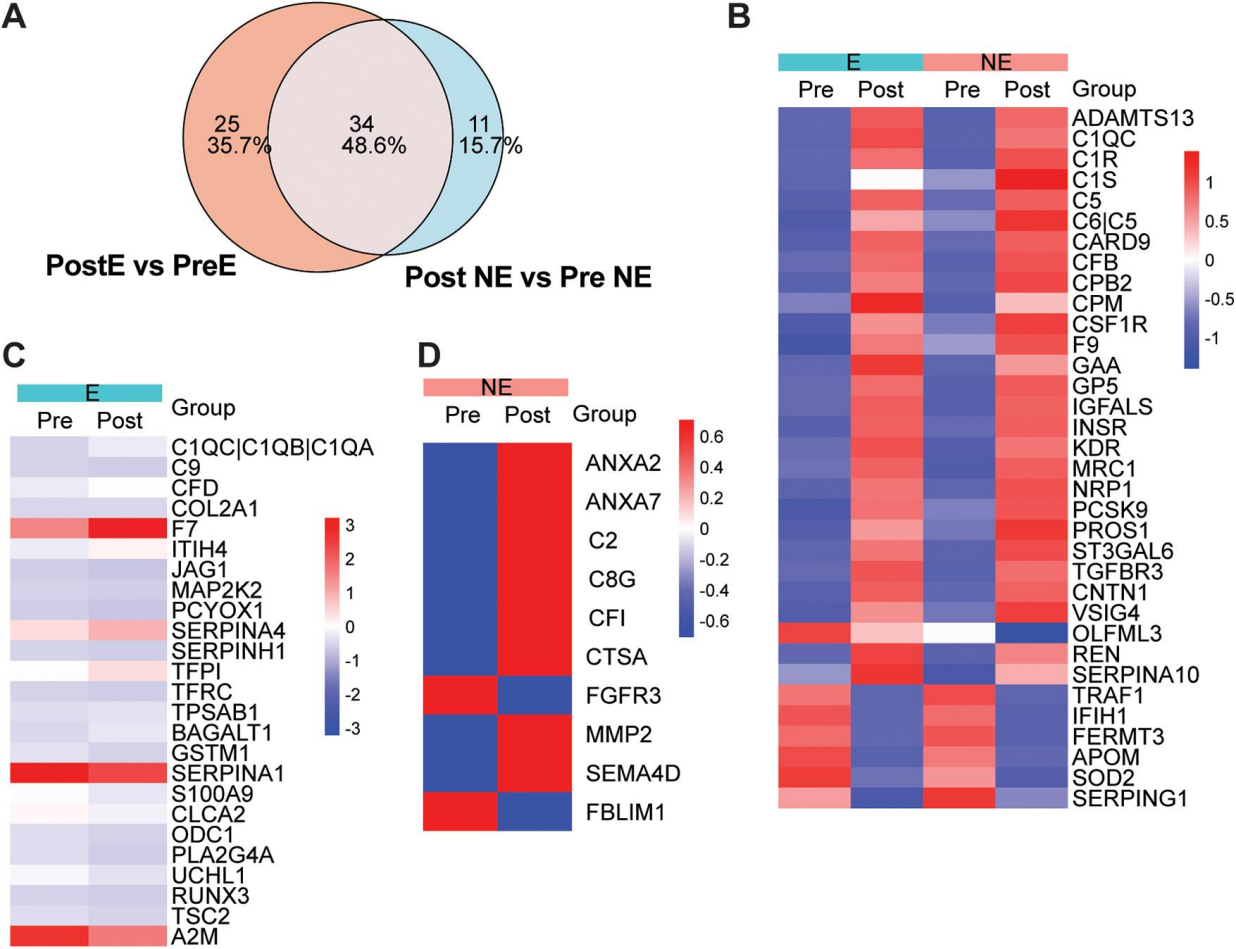
Venn diagram of differentially expressed proteins in exercise and no-exercise PCC groups. **(A)** Venn diagram comparing significantly altered proteins between post-exercise vs. pre-exercise (post-E vs. pre-E, red circle) and post-non-exercise vs. pre-non-exercise (post-NE vs. pre-NE, blue circle). A total of 34 proteins (48.6%) overlapped between the two groups, while 25 proteins (35.7%) were unique to the exercise group and 11 proteins (15.7%) were unique to the non-exercise group. **(B)** Heatmap of the 34 differentially expressed proteins that overlapped between the exercise and no-exercise groups; **(C)** Heatmap of proteins that were unique to the exercise group; **(D)** heatmap of proteins that were unique to the no-exercise group.

Among the differentially expressed proteins, 50% were modulated in a similar manner in both the exercise and no-exercise groups (Figures 2A,B). In addition, 25 and 11 proteins were specific to the exercise and no-exercise group, respectively (Figures 2A–D). In the exercise group, upregulated proteins included complement factors (C1QC, C1QA, C1QB, C1R, C1S, C5, C6, C9, CFD, CFB), coagulation-related proteins (F7, F9, PROS1, CPB2, TFPI), metabolic regulators (PCSK9, GAA, TFRC, INSR), and extracellular matrix or signaling molecules (ITIH4, JAG1, KDR, TGFBR3, OLFML3). In contrast, downregulated proteins included stress and inflammatory mediators (S100A8, TRAF1, IFIH1, FERMT3), antioxidant enzymes (SOD2, GSTM1), transport/metabolic proteins (APOM, ODC1, PLA2G4A, UCHL1, TSC2), and protease regulators (SERPINA1, SERPING1, A2M). This distribution demonstrates that exercise drives both activation and suppression of distinct proteomic signatures, highlighting remodeling of immune, vascular, and oxidative stress pathways.

**Figure 3:**
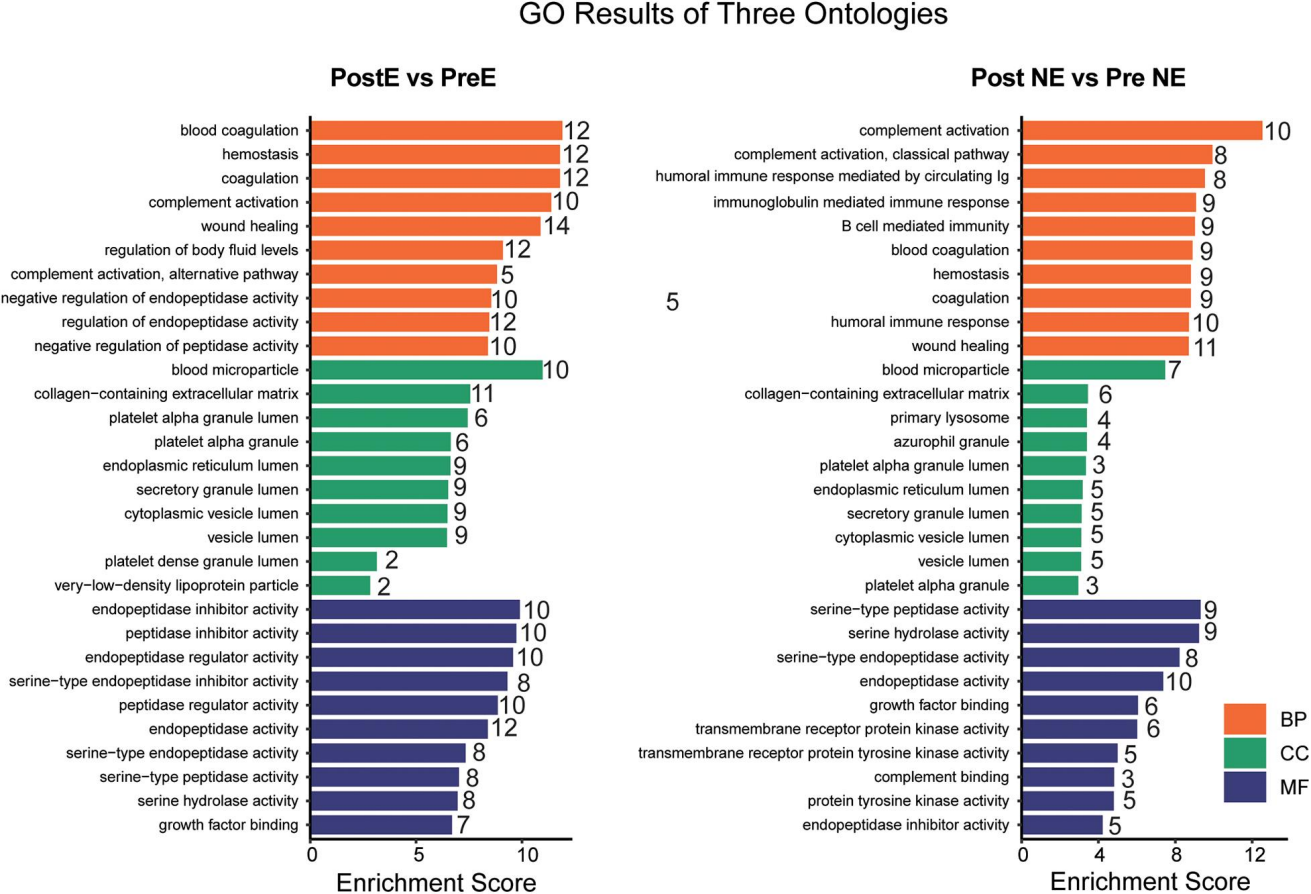
Gene ontology (GO) enrichment of the differentially expressed proteins. The number of proteins identified is noted for each column.

Gene Ontology enrichment analysis of the differentially expressed proteins revealed significant associations with biological processes that showed some overlap as well as differences between the exercise and no-exercise groups (Figure 3). The most enriched categories in the exercise group included complement activation by the alternate pathway, regulation of body fluid levels and negative regulation of peptidase and endopeptidase activity suggesting a fine-tuned protease–antiprotease balance after exercise. These biological processes collectively point to improved regulation of vascular integrity in PCC individuals following the exercise intervention. In the no-exercise group, immune responses appear to be altered over time.

## 4 Discussion

In this study we analyzed the plasma for proteins using aptamer technology in samples obtained from individuals who went through a tailored exercise regimen described previously (13). In our previous study using LS-MS followed by DIA-NN analyses, we had identified a handful of proteins out of 6,000 + proteins that were identified. In this study using SomaLogic platform, we were able to identify additional proteins from a predetermined list of 1,500 proteins, that were modulated following tailored exercise program. Various studies have compared the two platforms in detail and have identified the strengths, complementarity and the uniqueness of each of the different approaches to study plasma proteome (20). Affinity based proteomics platforms (e.g., SomaLogic and Olink) provide high-throughput, multiplexed quantification of pre-determined and hence biased, low-abundance proteins, while LC-MS provides broad, unbiased discovery of proteins by measuring peptides, offering deep proteome coverage for unbiased biomarker discovery.

The ability of SomaScan to detect low abundance proteins is evident from the identification of multiple proteins that were differentially regulated over time with or without intervention in pathways that have been shown to be associated with PCC in other studies [https://covimapp.serve.scilifelab.se/; (21)]. Despite the low sample numbers, fold changes in these proteins were significant following tailored exercise intervention. While it is not possible to come to definite conclusions from the observed differences, this study provides proof-of-principle to further analyze the changes in the plasma proteome following a mild physical activity regimen that is beneficial in individuals living with PCC or any other chronic condition to understand the underlying pathology.

Limitations of this study: Some of the major limitations of this study is the small sample size, absence of stratification of PCC groups based on symptoms, sex and age, and restricted numbers of proteins analyzed. Nonetheless, the symptom-titrated physical exercise has shown promise in improving the quality of life in selected individuals living with PCC (22–30), and understanding the changes in the proteomics can provide clues on the pathways affected.

## Data Availability

The original contributions presented in the study are included in the article/Supplementary Material, further inquiries can be directed to the corresponding author.
